# Saudi national survey of point -of -care ultrasound training in anesthesiology residency programs

**DOI:** 10.1186/s12871-026-03684-4

**Published:** 2026-02-13

**Authors:** Rothana Majid Aljehani, Abdulrahman Alboog, Haneen Alnazzawi, Albaraa Alnazzawi, Razan Altumaihi, Omar Addas

**Affiliations:** 1 Division of Anesthesiology, Department of Surgery, College of Medicine, University of Jeddah, Hamzah Ibn Al Qasim St, Al Sharafeyah, Jeddah, 23218 Saudi Arabia; 2 General Medicine Program, Batterjee Medical College, Jeddah, Saudi Arabia; 3 Department of Anesthesiology, International Medical Center, Jeddah, Saudi Arabia; 4 Department of Intensive Care Medicine, King Fahad General hospital, Jeddah, Saudi Arabia

**Keywords:** POCUS, Anesthesiology, Residency programs, Medical education, Saudi arabia

## Abstract

**Background:**

Point-of-care ultrasonography (POCUS) has emerged as a valuable tool in anesthesiology, enhancing procedure accuracy and clinical decision-making. Although its practice has gained momentum in Saudi Arabia, However, evidence regarding its integration into anesthesiology residency training remains limited.

**Methods:**

We conducted a national cross-sectional survey targeting program directors of anesthesiology residency programs accredited by the Saudi Commission for Health Specialties. The survey evaluated the current state of POCUS training, the assessment methods employed by each institution, and the perceived challenges to its instruction.

**Results:**

A total of 36 out of 42 program directors responded (85.7% response rate). All programs state that POCUS training is offered for vascular access, nerve blocks, neuraxial blocks, and TTE. However, 77.8% reported a lack of organized training programs.A total of 18 programs (50%) reported using formal evaluation methods. Faculty expertise was limited, with 83.3% of directors estimating that ≤ 25% of faculty were proficient in TEE, lung, and gastric ultrasound. Only 11 programs (30.6%) had a designated POCUS expert, and 66.7% reported no funding for extracurricular ultrasound training.

**Conclusions:**

POCUS training in Saudi anesthesiology residency programs is markedly underdeveloped; Implementing a standardized curriculum, enhancing faculty training, and increased institutional support to ensure residents achieve competency in core and advanced ultrasound applications.

## Background

Over the past decade, point-of-care ultrasound POCUS has become an important tool in many different areas of medicine, enhancing patient safety, making procedures more accurate, and making decisions faster [1]. Numerous studies in emergency medicine and primary care illustrate the value of ultrasonography in patient management [2]. Since the 1990s, emergency medicine departments in the United States have started the use of POCUS to aid in the assessment of trauma patients via the focused assessment with sonography for trauma (FAST) examination [3]. Recently, the scope of clinical ultrasound (US) applications increased to incorporate nearly all medical specialties [4]. POCUS has become increasingly important in the field of anesthesia, as it enhances perioperative patient management, procedural guidance, and diagnostics. It is emerging as a critical and core skill that anesthesiologists should possess [5]. Common application examples of POCUS in anesthesiology include ultrasound-guided nerve block, vascular access, airway ultrasound, gastric ultrasound, lung ultrasound, neuraxial sonography, focused transthoracic (TTE) and transesophageal echocardiography (TEE). Thus, there are multiple potential areas where ultrasound can play a significant role in guiding and improving the safety and efficacy of many interventions [6, 7]. Currently, Saudi Arabian anesthesiology residency programs lack published data describing the incorporation of POCUS teaching and specific applications. Therefore, this study aims to assess the extent of POCUS integration in anesthesiology residency programs in Saudi Arabia, identify key barriers to implementation, and highlight opportunities to improve ultrasound education in alignment with international standards.

## Methods

Ethical approval was granted by the Institutional Review Board at University of Jeddah Institutional (no. HAP-02-J-094 on December 1, 2024). All participants provided electronically signed informed consent prior to participating in the survey. No identifying personal data were collected, and responses remained anonymous.

We identified 42 anesthesia residency programs in Saudi Arabia accredited by the Saudi Commission for Health Specialties and invited all program directors to participate in the survey. A structured survey consisting of 20 questions was developed using Google Forms and distributed electronically among program directors between December 2024 and January 2025. The survey link was shared through WhatsApp, followed by reminders every two weeks subsequently for a total of four reminders. The questionnaire was adapted from a previously published study by Mok et al. [4] with modifications made to suit the Saudi context and study objectives. Prior to finalization of the survey, a draft version was reviewed by five anesthesiology program directors for expert feedback and content clarity. The questionnaire covered multiple domains such as current training and evaluation in POCUS, faculty expertise, perceived challenges, and future directions. Definitions were standardized: a structured training program was defined as a formalized training curriculum with a schedule of instruction and learning objectives. A Formal evaluation plan was defined as any systematic approach used to evaluate resident competence, such as procedure logs, checklists, written or practical exams, or image review.

Survey data were securely stored on the Google Forms server and downloaded for analysis following data collection. Descriptive statistics (frequencies, means, and percentages) were calculated using Microsoft^®^ Excel (version 16.103.2). No inferential statistical analyses were conducted.

## Results

**Fig. 1:**
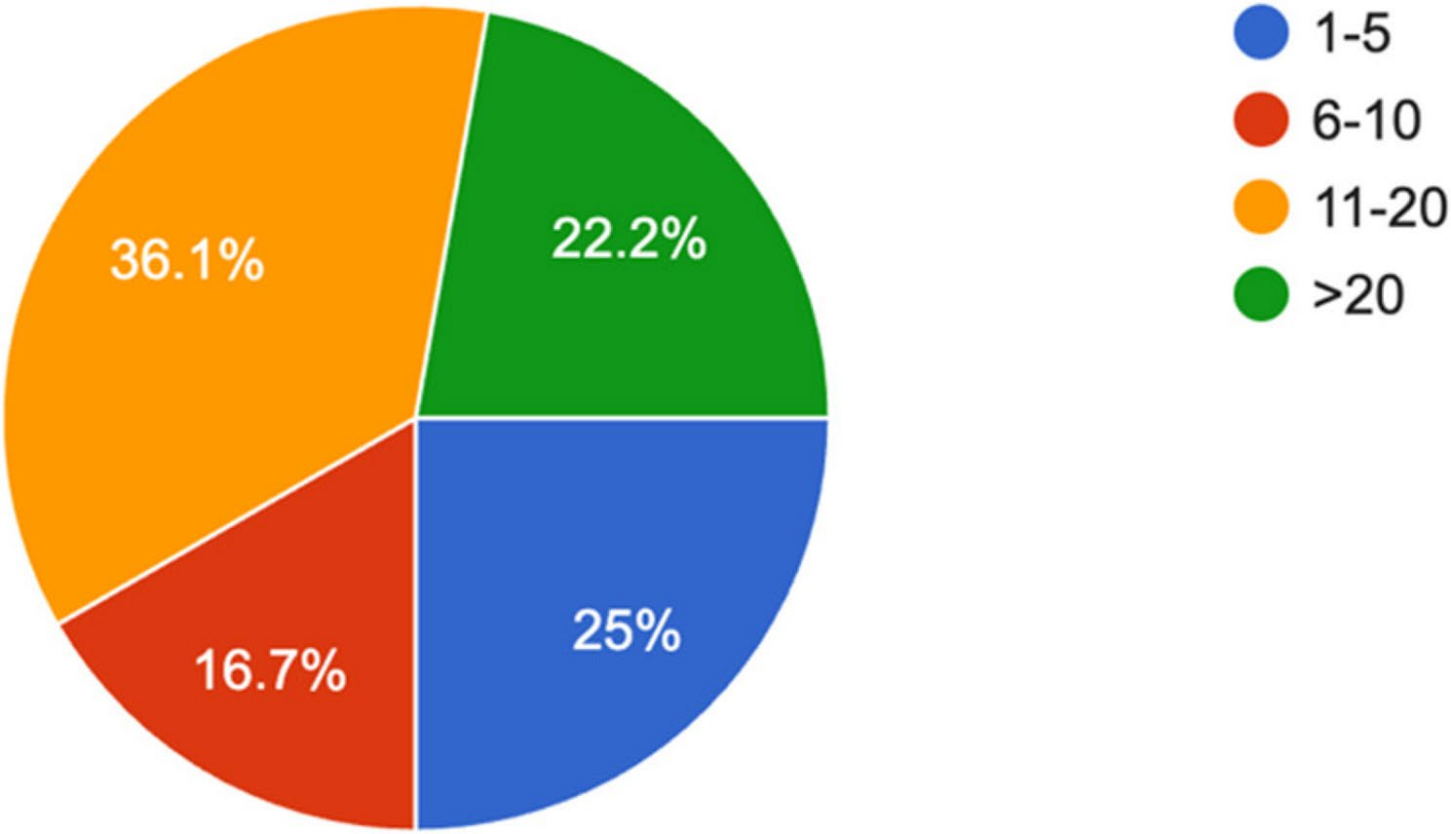
Number of residents in respected programs

A total Thirty-six out of forty -two program directors of anesthesia residency programs completed the survey, yielding a response rate of 85.7%. Regarding number of residents enrolled in residency programs, 13 program directors reported having 11–20 residents (36.1%), while 9 programs (25%) had between 1 and 5 residents (Fig. 1).

**Fig. 2:**
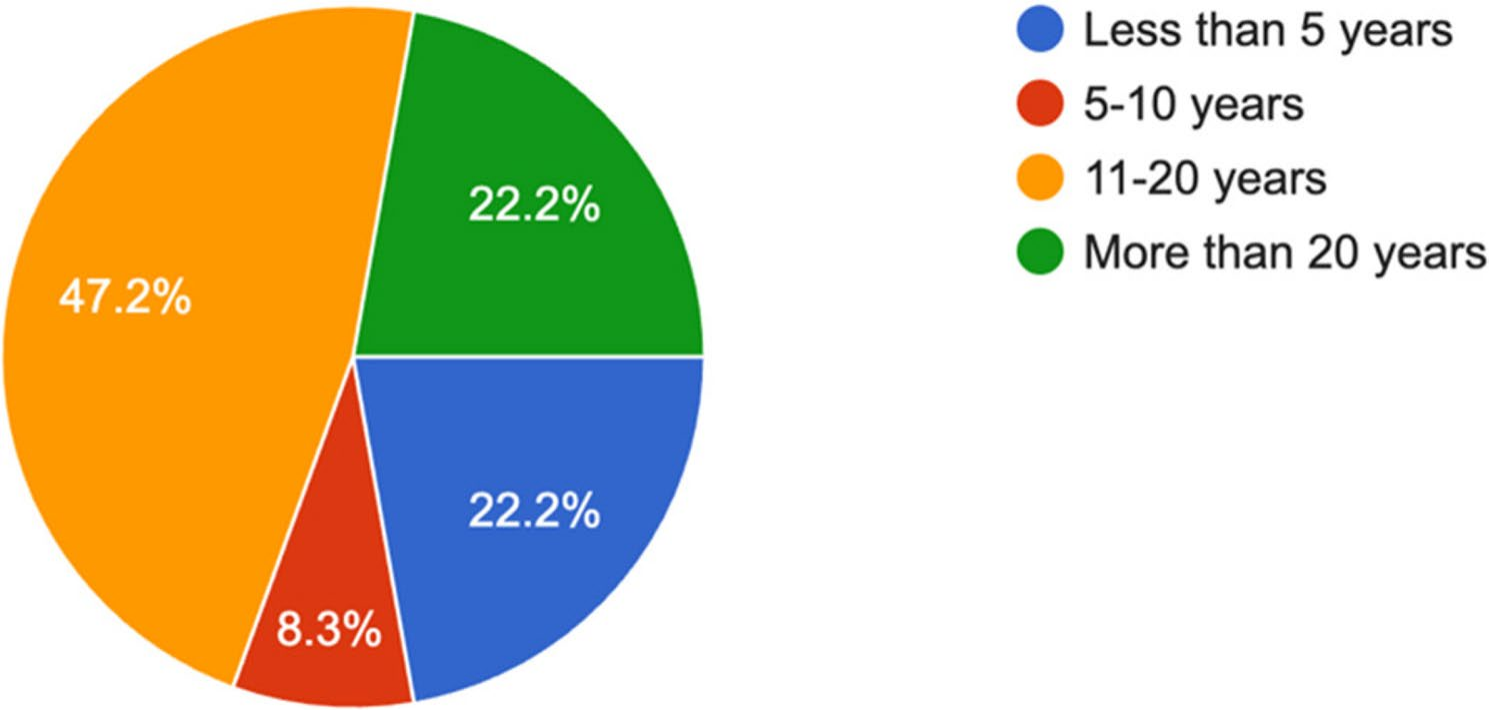
Number of years of experience

Regarding anesthesia experience, most program directors (*n* = 20, 55.6%) had 11 to 20 years of professional experience, and the majority (*n* = 28, 77.8%) held consultant-level positions, while only 22.2% had more than 20 years of experience. More than half were trained locally via the Saudi Commission for Health Specialties (SCFHS), while 36.1% trained abroad (Fig. 2).

**Fig. 3:**
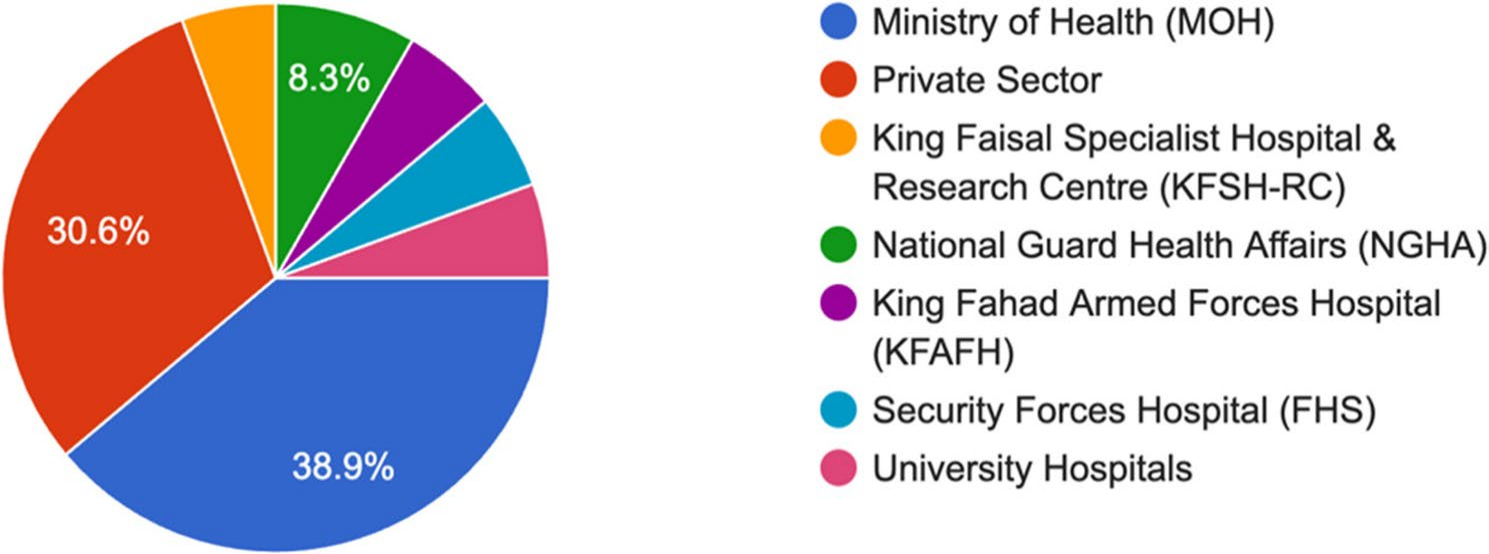
Resident’s training centers

Geographically, the responses came from the Western region (*n* = 15, 41.7%), followed by the Eastern (*n* = 9, 25%) and Central regions (*n* = 8, 22.2%). In terms of the type of facilities that participated, 38.9% were from the Ministry of Health, and 30.6% were from the private sector (Fig. 3).

**Fig. 4:**
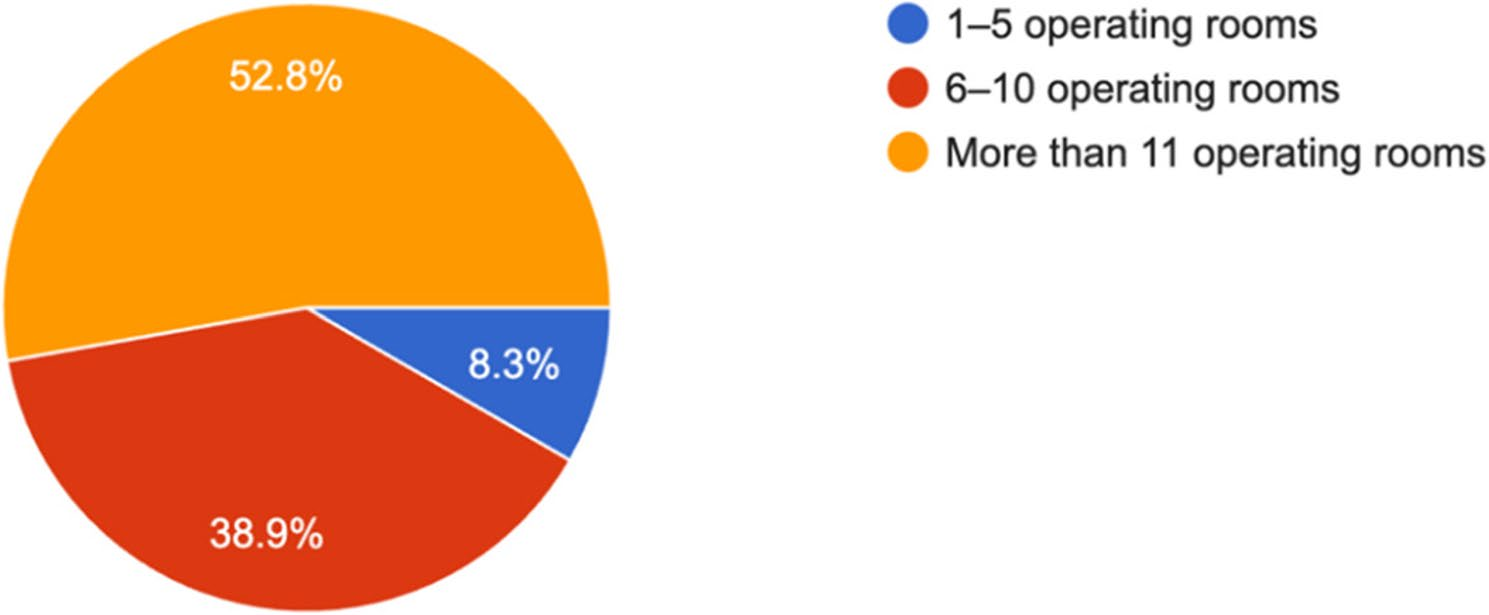
Operating Rooms in respected Centers

Also, most institutions in our study reported having more than ten operation rooms (52.8%), while 38.9% had between six and ten operating rooms (Fig. 4).

**Fig. 5:**
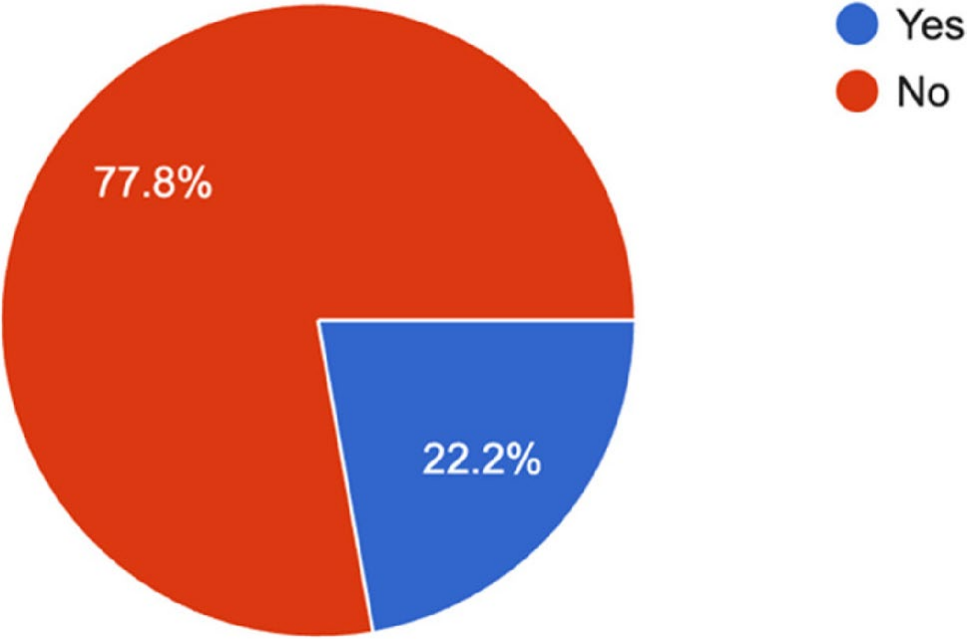
Formal training in POCUS

All programs (100%) reported providing POCUS training for vascular and arterial access, peripheral nerve blocks, neuraxial blocks, and transthoracic echocardiography (TTE). However, 28 programs (77.8%) lacked a structured training curriculum (Fig. 5).

**Fig. 6:**
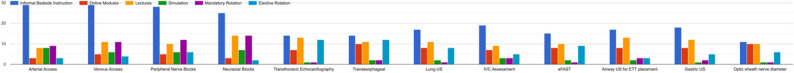
Training modalities used to teach residents

Informal bedside teaching was the most common method of instruction, while 33 programs (91.7%) also provided lectures by faculty members. Online modules (*n* = 28, 77.8%) and simulation sessions (*n* = 26, 72.2%) were frequently used. Formal clinical POCUS rotation was offered in only 9 programs (25%) (Fig. 6).

**Fig. 7:**
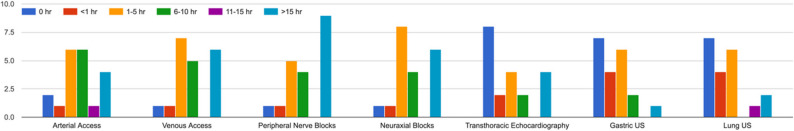
The amount of formal training (*in hours*) that residents may receive by the end of residency

Although the SCFHS Anesthesiology Curriculum currently lacks specific requirements regarding minimum hours or expected number of scans by the end of residency training, most program directors indicated the necessity for structured training, especially peripheral nerve blocks. Eight programs specifically reported requiring at least 15 h of formal training for peripheral nerve blocks, although no specific requirement was mentioned regarding the number of scans performed during residency rotations (Fig. 7).

**Fig. 8:**
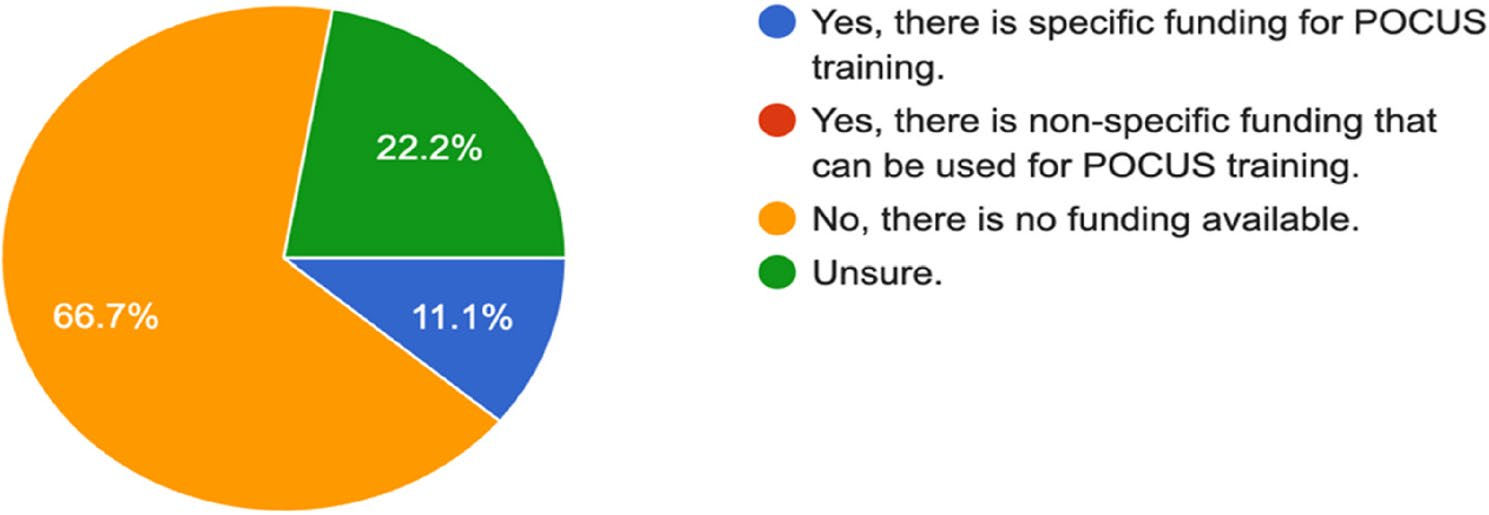
Funding availability for POCUS

Program directors were inquiring about the availability of funding for extracurricular POCUS training, such as POCUS workshops offered during conferences or by other academic institutions; 66.7% reported a lack of funding for such activities, and only 11.1% indicated that funding for POCUS is available, while the remaining are uncertain about the availability of financial support (Fig. 8).

Among the programs that reported using formal assessment methods (*n* = 18), all of them (100%) utilized direct observation and supervision as the primary evaluation tool. Over half (*n* = 10, 55.6%) required residents to complete a minimum number of scans or procedures.

**Fig. 9:**
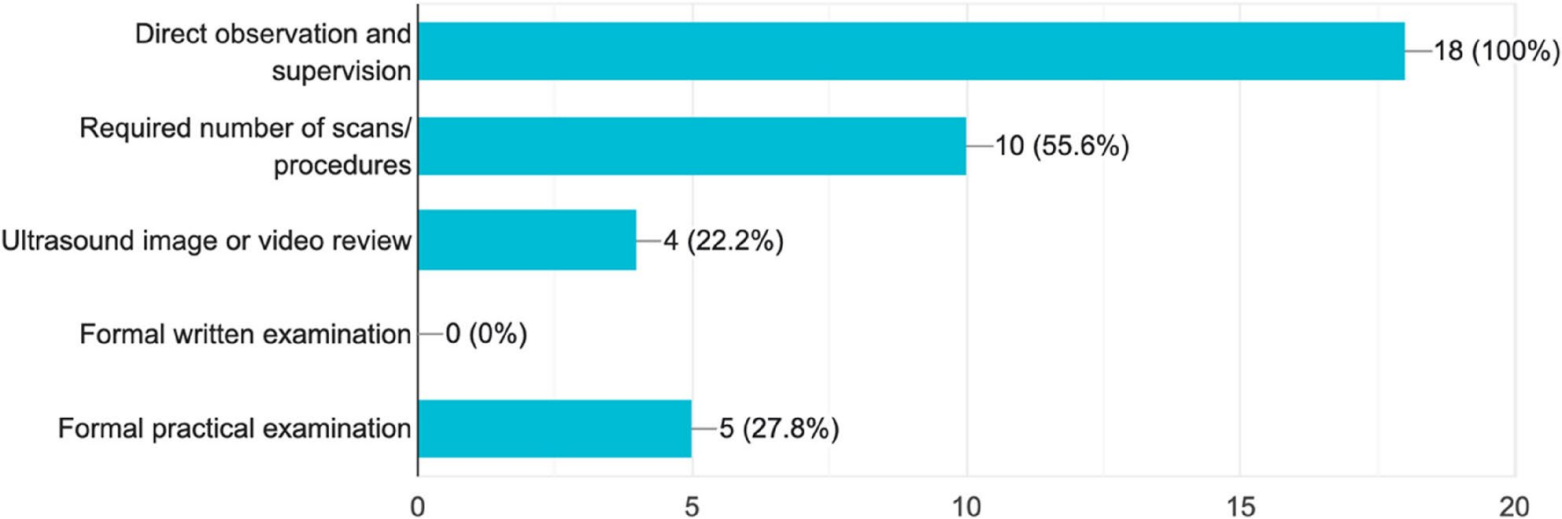
Modalities of formal assessment

Less commonly used methods included ultrasound image or video review (*n* = 4, 22.2%) and formal practical examinations (*n* = 5, 27.8%). Notably, none of the programs reported the use of formal written examinations (0%) (Fig. 9).

Program directors were asked to assess the importance of Anesthesiology residents being competent in various POCUS applications at the end of their training. In this context, competence was considered as the ability to independently obtain and interpret ultrasound imaging to answer a focused clinical question or to facilitate a procedure.

**Fig. 10:**
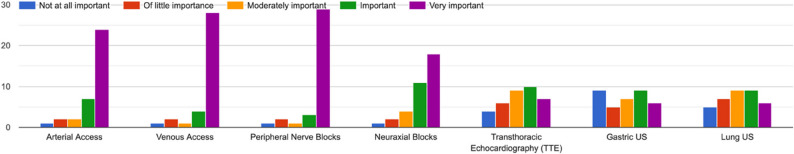
Rate the importance of trainees achieving competence

The majority (≥ 89%) rated competence in vascular access and peripheral nerve blocks as “important” or “very important.” In contrast, only 47% considered TTE competency essential. Notably, none of the program directors added any applications that were not listed on the survey that were considered essential for graduating residents (Fig. 10).

**Fig. 11:**
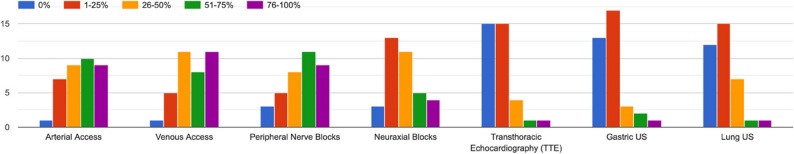
Competency of anesthesiologists in different pocus modalities in their respected centers

Program directors were also asked to estimate the percentage of faculty anesthesiologists within their training program who are competent in the POCUS applications listed in our survey. The proportion of faculty estimated to be competent in these applications varied widely among programs. Competency across programs was perceived to be relatively high in vascular access and in peripheral nerve blocks and estimates for a given program usually ranged from 50% to over 75%. On the other hand, a remarkable 83.3% of program directors estimated that their faculty is 25% or less competent in the skills of TTE, lung ultrasound, and gastric ultrasound, reflecting a serious gap in expertise in advanced applications (Fig. 11).

At the end of the survey, only 11 programs (30.6%) reported having a designated POCUS expert.

**Fig. 12:**
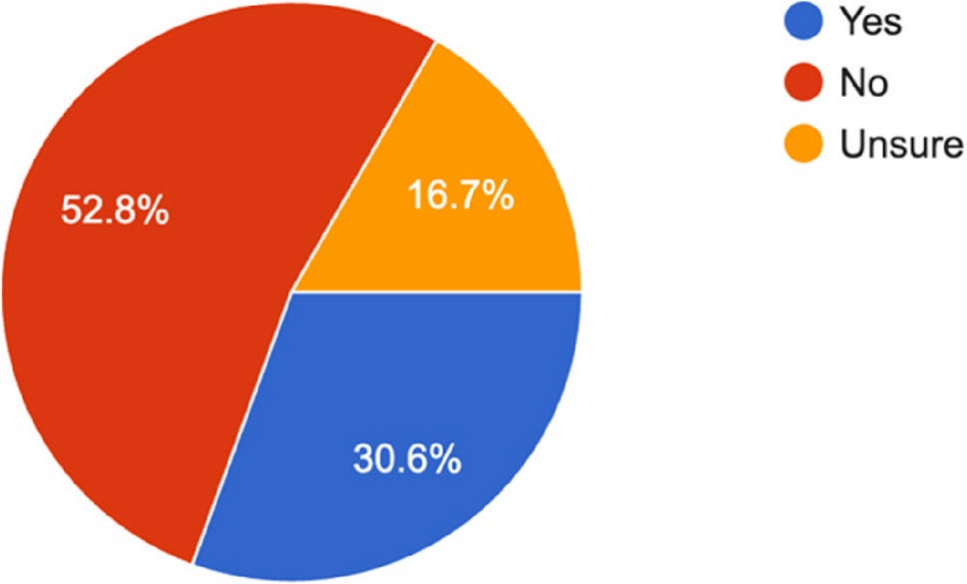
Anesthesiologist in your program who acts as the local POCUS expert

These results emphasize critical gaps in faculty expertise and program leadership that may adversely impact comprehensive POCUS training within Anesthesiology residency programs (Fig. 12).

## Discussion

POCUS, or point-of-care ultrasound, is an advanced portable diagnostic tool with several applications readily accessible at the patient’s bedside. The use of POCUS has surged in the last ten years across several medical fields, enabling improved patient diagnosis, evaluation, and management [8]. Over the past decade, POCUS has been extensively used across several medical specialties in Saudi Arabia, particularly in emergency medicine and critical care [9, 10]. However, its incorporation into residency training and clinical practice remains variable and underdeveloped, as prior survey studies in Saudi Arabia show that utilization of POCUS is hindered by training gaps, lack of standardized curriculum requirements, and limited resources [11]. In anesthesiology, POCUS has emerged as a vital competency, enhancing perioperative safety, procedural precision, and clinical decision-making, especially in critical and time-sensitive scenarios. Although its recognized value is acknowledged worldwide [12–14], anesthesiology has been slower to incorporate POCUS into routine training and practice, highlighting a significant educational gap [15]. This national survey provides a comprehensive overview of the current status of point-of-care ultrasound (POCUS) training within anesthesiology residency programs in Saudi Arabia. Our results reveal a significant opportunity for improved curriculum development, a perspective supported in an international context. Specifically, our data aligned with a study conducted in Canada in 2017, which also reported broader implementation of POCUS in emergency medicine and critical care compared to anesthesiology [4]. This persistent global trend reflects a common discipline-specific issue that extends beyond regional limitations, perhaps involving factors such as traditional training methods and uneven resource allocation. However, in comparison with the finding of Daniel Mok [4], important differences emerged that highlight unique characteristics of our national study.

Initially, our study achieved a higher number of responses from program directors, potentially providing a more comprehensive representation of the national training. Furthermore, while Canadian programs reported more consistent integration of transesophageal echocardiography (TEE), our data show that TEE is less routinely incorporated into Saudi anesthesiology residency programs. This trend of variable and often restricted POCUS training is not unique to Canada. Our findings are further supported by a similar survey conducted in the United States [16], which also reported considerable variability in POCUS training among anesthesiology residency programs. Surprisingly, the similarity in both the United States survey and our survey was that training was most comprehensive for basic competencies like vascular access and peripheral nerve block, while advanced applications such as transthoracic echocardiography, transesophageal echocardiography, gastric ultrasound, and lung ultrasound were less often included in teaching. The similarities extend to the recognized barriers, as US programs also identify the main barriers as lack of faculty expertise and standardized curricula. Also unsurprisingly, many programs rely on informal bedside teaching without structured competency-based assessments.

The critical implication of these shared barriers is represented in our data, where 28 out of 36 program directors (77.8%) reported having no formal POCUS training themselves. Indeed, this represents a potential cyclical problem whereby a lack of formally trained faculty propagates into a lack of formal training for residents. Encouragingly, previous promising pilot studies, such as one performed by Lee et al. [17] in the U.S., have demonstrated a structured POCUS curriculum can heavily improve both resident confidence and measured competence. Such findings support our conclusion that well-designed, standardized training programs could address these educational deficiencies.

Apart from curricular challenges, financial constraints regarding POCUS training were noted as a major obstacle in our data. About 66.7% of program directors cited a lack of financial support for POCUS-related resources. Without adequate funding, programs may not be able to purchase the necessary ultrasound equipment, embed simulation-based sessions, or cover support to attend POCUS-related workshops. These further restrain residency programs from implementing comprehensive training. Overall, our results again underline the competence of POCUS, which residents in anesthesiology should possess. However, generally poor curricula, lack of faculty training, and lack of funding present major obstacles to its implementation. Overcoming these deficiencies will require national standardization efforts—investing in faculty development and increasing resources that allow long-term implementation in residency training in Saudi Arabia.

### Barriers of integration

Despite its numerous benefits, POCUS integration into anesthesiology training continues to face several challenges in Saudi Arabia, such as lack of awareness and limited access to POCUS-specific funding, which discourages many residents from participating in POCUS-related courses. Also, there is a shortage of qualified expertise in POCUS, and in this way, in-house education by experienced instructors becomes quite difficult to perform within residency programs.Time constraints and heavy workloads also restrict residents’ ability to participate in supplementary training. Additionally, while one of the most important modes of learning is through experience, many institutions lack the necessary equipment, such as working ultrasound machines, sterile probe covers, or procedural equipment like spinal needles, to facilitate this kind of learning. Psychological factors also have their contributions to make. The fear of failure or rejection and the limited time available in some clinical rotations create an obstacle to residents’ behavior of accepting POCUS training or putting their knowledge into practice. This landscape of multifactorial barriers creates a vicious cycle where, because of limited resources and faculty, there is reduced resident exposure, leading eventually to a problem with clinical competency.As POCUS is gaining more attention in anesthesiology, the solution to these barriers will require persistence on the part of education, faculty development, and institutional investment. Full implementation of a standardized, comprehensive training framework requires significant resources to realize the full clinical potential of POCUS.

### Limitation

This study has a number of limitations that are inherent in its design and methodology. Being an online survey, the results are prone to response bias and the possibility of the respondents misinterpreting the questions. The use of the program directors’ perspective also has the potential for social desirability bias, where the respondents may view their programs more positively. Additionally, the study did not use an objective assessment of the curricula, faculty abilities, or resident performance, which would have allowed it to link practices with actual educational outcomes. There is also the possibility of non-response bias, where the characteristics of the non-responding programs may systematically differ from those that responded. Lastly, the study did not use the point of view of the residents, which would have allowed the generalizability of the results regarding trainee experiences and satisfaction with POCUS training.

## Conclusions

The current study provides a national perspective on the current status of point-of-care ultrasound training in anesthesiology residency programs in Saudi Arabia. While all programs offer basic ultrasound exposure, challenges to implementation exist due to the lack of training curricula, faculty expertise, and funding. The results of the study indicate that there must be training infrastructure, faculty development programs, and funding to ensure high-quality training in ultrasound. National coordination and planning may assist in overcoming these challenges and incorporating POCUS training into the core competency of anesthesiology.

## Data Availability

The survey was administered through Google Forms. No personally identifying information was collected. All responses were anonymized at the time of collection and securely stored. Data were downloaded to a password-protected institutional device for analysis, and access was limited to the study team.The datasets generated and analyzed during the current study are not publicly available due to confidentiality agreements with participating institutions but are available from the corresponding author on reasonable request. Requests for data access should be directed to Dr. Rothana Aljehani at rmaljehani@uj.edu.sa.

## Abbreviations

POCUS: Point–of–care ultrasonography
TTE: Transthoracic echocardiogram
US: Ultrasound
FAST: Focused assessment with sonography for trauma
TEE: Transesophageal echocardiography
IVC: Inferior vena cava
Efast: Extended Focused Assessment with Sonography for Trauma
ETT: Endotracheal tube
SCFHS: Saudi commission for Health Specialties
